# Desmoid Fibromatosis Fused With a Lipoma in the Upper Arm

**DOI:** 10.7759/cureus.55430

**Published:** 2024-03-03

**Authors:** Frank Traub, Tobias De Jager, Ulf K Hofmann, George Farah, Saskia M Sachsenmaier

**Affiliations:** 1 Orthopaedics and Traumatology, University Medical Center of Johannes Gutenberg University Mainz, Mainz, DEU; 2 Orthopaedic Surgery, Eberhard Karls University of Tübingen, Tübingen, DEU

**Keywords:** multidisciplinary collaboration, mixed connective tissue tumors, musculoskeletal system, fused tumor, lipoma, desmoid fibromatosis

## Abstract

Lipoma, the most common mesenchymal tumor, often appears as a slow-growing mass in the musculoskeletal system (MSK). While generally non-invasive, their location can cause symptoms. Desmoid fibromatosis (DF), a rare and locally aggressive neoplasm, poses challenges in MSK system diagnosis and management due to its infiltrative nature. Despite lacking metastatic potential, DF has a high recurrence rate, classifying it as "intermediate, locally aggressive" in the WHO classification. Collaborative efforts among orthopedic surgeons, radiologists, and pathologists are crucial for accurate diagnosis and treatment planning for all tumors of the MSK system. This case report presents the first documented example of a DF within a lipoma, highlighting the challenges of diagnosing and treating musculoskeletal tumors.

## Introduction

Desmoid tumor (from the Greek' desmos' meaning tendon-like), also known as aggressive fibromatosis or desmoid fibromatosis (DF), is a very rare myofibroblastic mesenchymal neoplasm [1-3]. The tumor is characterized by local infiltration with a high incidence of recurrence but lacks the potential of metastasis [1], categorizing desmoids as 'semi-malignant' [3]. DF is traditionally classified into intra-abdominal types, often linked with familial adenomatous polyposis (FAP). It can also be categorized as abdominal, commonly observed in women of reproductive age following pregnancy, or extra-abdominal, typically manifesting as a pelvic or shoulder girdle tumor [2], as in our case report.

Desmoid-type fibromatosis accounts for 0.03% of all neoplasms and 3% of soft-tissue tumors [1]. It occurs mostly sporadically. However, pathogenesis is associated with adenomatous polyposis coli or pre-existing Gardner's fibroma. Blunt trauma or previous surgery seems to be associated with tumor growth, which is also regulated by several factors, including steroidal sex hormones [4]. Histopathologically, desmoid tumors usually show an infiltrative border composed of myofibroblasts within a collagenous stroma and lack a true capsule.

Most show distinct nuclear accumulations of beta-catenin, which has a specific immunohistochemistry diagnostic feature. In approximately 50-85% of sporadic desmoid tumors, a mutation of the CTNNB1 gene leads to the overproduction of beta-catenin [1,2]. MRI is the imaging method of choice. It provides the optimum diagnostic method for primary tumor evaluation, as well as for postoperative monitoring. However, tissue confirmation for a final diagnosis is required. There are no compulsory guidelines for patients regarding the frequency of check-ups before or after treatment [5]. Still, follow-up imaging is generally performed for 3-6 months for the first year and 6-12 months up to the 5th year [1].

Conversely, a lipoma is a typical lesion that accounts for approximately 16% of soft tissue mesenchymal tumors [6]. They are defined as benign neoplasms of mature adipocytes (white fat cells) and are not distinguishable histologically from normal fat [7,8]. Intramuscular lipoma is most common within large muscles of the thigh, upper arm, and shoulder [6]. Lipomas are categorized by anatomic location as either superficial (subcutaneous) or deep [8]. Approximately 80% are less than 5cm in size and superficial [8], whereas deep lipomas are less common [9]. The etiology of lipomas still needs to be understood entirely. Risk factors include family history, obesity, and inactivity [10]. Lesions usually present as asymptomatic, painless soft tissue masses and may enlarge slowly [9,11]. Clinical symptoms are uncommon but include local pain, tenderness, and nerve compression [8]. Radiologic evaluation is confirmatory in up to 71% of cases, especially magnetic resonance imaging (MRI) [12]. Since lipomas are benign tumors, treatment is usually unnecessary [10]. Surgical removal of a lipoma should be performed if the tumor is causing pain, restricting movement, or causing aesthetic impairment.

## Case presentation

We report on a 75-year-old male patient who attended our tumor consultation service showing a solid lesion on the right upper limb, which was progressively enlarging for a few months. An MRI, which was arranged a few weeks earlier on the advice of his family doctor to rule out a sarcoma, reported an indeterminate tumor with a recommendation for further pathological clarification.

The patient's medical history revealed no pain or immobility; only night sweats were reported without weight loss or fatigue was mentioned. A positive family history included a mother with a brain tumor and a brother with colon cancer. Physical examination showed normal sensation and movement of the arm and ruled out swelling of axillary, infra- or supraclavicular lymph nodes.

**Figure 1:**
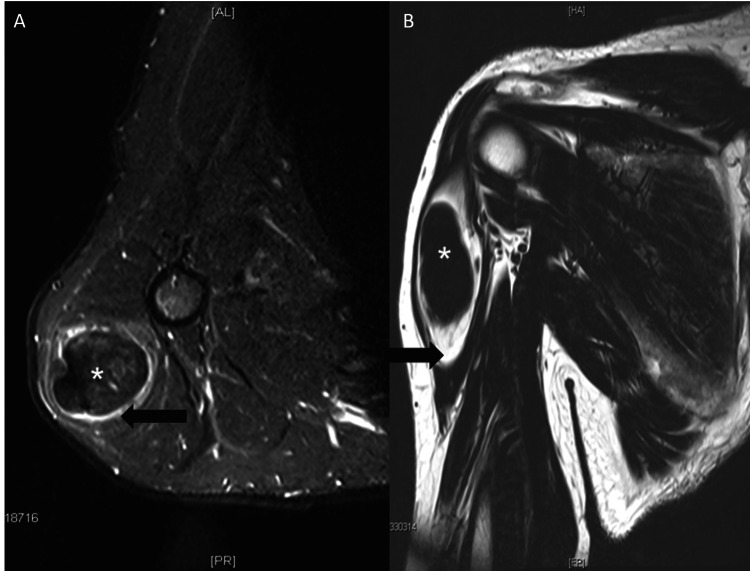
A) Axial T2 and B) coronal T1 MRI of the right shoulder girdle showing extra-abdominal fibromatosis (*) inside a lipomatous tumor (arrow) in the deltoid muscle.

The patient then underwent a CT scan to determine the status of his lungs and rule out any bony involvement of the tumor. The radiologist reported a suspicious nodule in the left upper lobe and no evidence of bone infiltration (Figure 1). An incisional biopsy of the central tumor aspect was performed as the next diagnostic step.

On histology, the central tumor consists of elongated spindle-shaped fibroblasts arranged in a fascicular pattern with a prominent collagenous background. The tumor cells have poorly defined cell borders merging with the collagenous matrix. The nuclei are oval-shaped with little variations. The tumor contains mildly dilated blood vessels, and myxoid changes were present. Immunohistochemistry showed an accumulation of ß-catenin proteins in the nucleus. This led to further molecular diagnostics, and a T41A mutation of ß-catenin was detected, confirming the diagnosis of a desmoid-type fibromatosis.

At the next multidisciplinary sarcoma board meeting, further lesion management was discussed, with either surgery, radiotherapy, or watch and wait being considered. Primary resection of the right upper extremity lesion was recommended, and the patient agreed to have the tumor removed. 

**Figure 2:**
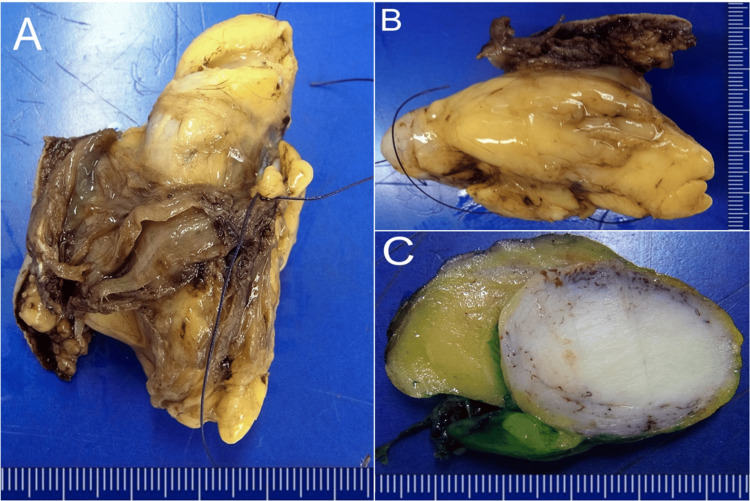
Macroscopic findings: A) Pathological specimen after en-bloc resection. B) The capsule of the lipoma is clearly visible. C) The dissected tumor reveals the pronounced grey-whitish swirled nodule within dense fatty tissue enclosed by a thin capsule.

The patient remained asymptomatic, and en-bloc resection of the tumor was performed one month later. The operative report stated a capsulized lipomatous tumor in the deltoid muscle. The tumor was bluntly dissected from the surrounding tissue. There were no documented postoperative complications; the drain was removed 36 hours after the operation. A physical therapist supervised the functional rehabilitation of the operated limb. The patient was discharged from the hospital two days after surgery. The specimen, weighing 88 g and measuring 10.5 x 5.3 x 5.5 cm, appeared to be made of skin, muscle, and fatty tissue. After splitting the specimen in half, a pronounced rough nodule appeared in the center of the specimen, surrounded by a fatty tissue tumor, which in turn was enclosed by a thin capsule (Figure 2).

**Figure 3:**
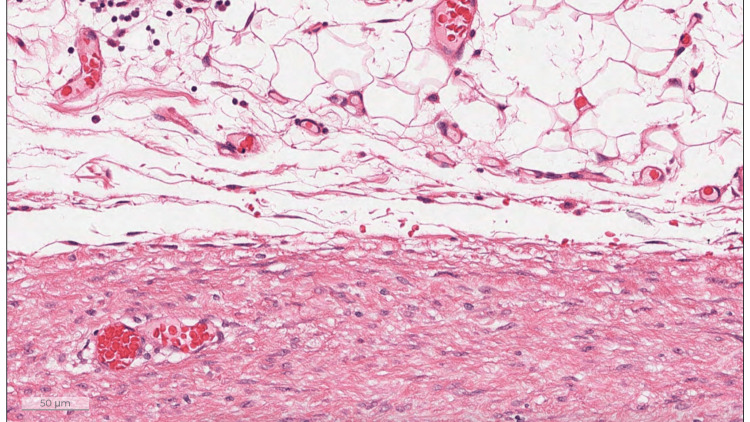
Histology shows fibroblasts arranged in a fascicular pattern with a prominent collagenous background. The tumor cells have poorly defined cell borders merging with the collagenous matrix. A dilated blood vessel is present. The fatty tissue is separated by a thin capsule, which appears somewhat detached here. The cells are arranged in lobules with thin fibrous septa. The fat cells show the typical configuration of a signet ring.

Microscopic examination of the central tumor showed a similar picture to that already described in the biopsy. The surrounding fatty tissue was arranged in lobules with thin fibrous septa. The cells showed uniform and had a signet-ring appearance. The absence of cellular atypia or mitotic activity suggested a benign adipose tumor (Figure 3). Fluorescence in situ hybridization (FISH) analysis revealed no amplification of MDM2 in either the lipomatous or fibromatous tumor cells, conclusively eliminating the potential diagnosis of an atypical lipoma or liposarcoma. Notably, this combination of pathological features appears unique, with no similar cases reported in the literature.

Ten days after surgery, the patient attended our tumor consultation for follow-up. He reported no postoperative discomfort and demonstrated unrestricted movement of the arm. Follow-up examinations, including MRI scans of the upper arm, showed no evidence of recurrence or functional limitations at six months, one year, and two years postoperatively.

## Discussion

The discovery of a fused tumor, specifically a semi-malignant desmoid fibromatosis within a lipoma, represents a unique clinical observation. This distinctive fusion of two distinct tumor entities within a lesion is a novel dimension to our understanding of tumorigenesis. Integrating information from radiology, pathology, and the surgeon is essential for a holistic understanding of the fused tumor. This multidisciplinary approach enhances diagnostic accuracy, refines treatment strategies, and ultimately contributes to improved patient outcomes by ensuring that the surgical intervention is well-informed and tailored to the unique characteristics of the lesion.

The principal clinical differential diagnosis of desmoid fibromatosis is a malignant soft-tissue sarcoma [13]. Following the guidelines, any soft-tissue sarcoma should trigger a referral to a multidisciplinary sarcoma center [1]. The optimal treatment and management of aggressive fibromatosis have not yet been defined [5,14]. More recently, a "watch and wait" policy has been advocated. In particular, extra-abdominal desmoids have a high risk of recurrence, suggesting more conservative management, including continued observation and eventually spontaneous regression of the indolent disease [1,5,14]. However, surgical excision with the additional radiotherapy option is still justified if negative margins are not achieved. In recent years, it has become increasingly apparent that thermal ablative therapies such as cryoablation and microwave ablation can also help control the symptoms of DF [15].

From a clinical perspective, the presence of a fused tumor involving semi-malignant desmoid fibromatosis within a lipoma requires a nuanced approach to diagnosis and treatment. The radiographical diagnosis of lipomas is critical for avoiding unnecessary surgery and the associated morbidity. At times, it may become necessary to operate on lipomas due to the local mass effect, and decision-making tools have been presented previously in the literature [16]. It should be noted that some experts recommend complete removal of lipomas when they exceed a size of 3.5 cm. This ensures that a possible liposarcoma is not missed [10]. With complete resection, recurrence of a lipoma is a rare finding (less than 1%) [6]. The decision to perform a complete surgical resection was made in order to confirm the diagnosis of a fused tumor. Due to the lipomatous capsule, the probability of incomplete resection was considered very low, and the risk of recurrence was therefore estimated to be negligible.

In general, there is no evident guidance on the frequency of re-evaluation for patients after treatment. Still, follow-up imaging is generally performed for 3-6 months for the first year and 6-12 months up to the 5th year [1].

The coexistence within the same tumor raises intriguing questions about the underlying molecular and genetic mechanisms that drive such an unusual fusion. Unraveling these intricate mechanisms could provide valuable insights into the overlapping pathways of tumorigenesis and potentially guide more targeted and effective therapeutic strategies.

## Conclusions

Identifying a fused tumor is a complex process that demands the expertise of radiologists, pathologists, and surgeons. Radiological imaging is critical for planning the surgery and providing detailed information about the tumor's location and structure. Pathological analysis is essential for accurate diagnosis and to guide treatment decisions. By working hand in hand, the surgeon can customize the surgical approach and adapt to any unforeseen findings during the surgery. This multidisciplinary approach significantly enhances diagnostic accuracy and optimizes treatment strategies, underscoring the importance of teamwork in addressing unique tumor presentations. Since this level of expertise is only available in specialized centers, it is imperative to refer patients with rare tutors to these centers.
